# Wireless sensing system for the welfare of sewer labourers

**DOI:** 10.1049/htl.2017.0017

**Published:** 2018-07-13

**Authors:** V.D. Ambeth Kumar, D. Elangovan, G. Gokul, J. Praveen Samuel, V.D. Ashok Kumar

**Affiliations:** 1 Computer Science and Engineering, Panimalar Engineering College, Chennai, 600123, India; 2 Electronics and Communication Engineering, Panimalar Engineering College, Chennai, 600123, India; 3 Computer Science and Engineering, St. Peter's University, Chennai, 600054, India

**Keywords:** gas sensors, wireless sensor networks, biomedical measurement, personnel, health hazards, industrial accidents, wireless sensing system, sewer labourer welfare, environmental pollution monitoring, toxic chemicals, toxic gases, microcontroller, internet of things, hazardous gases, heart beats, pulse detector, alert warning message, health center, first aid, fully automated, quick response time

## Abstract

There is a growing demand for the environmental pollution monitoring and control systems. In the view of ever increasing sources of toxic chemicals, these systems should have the facilities to detect and calibrate the source quickly. Toxic gases are the ones that cause health impact but humans are being exposed to it in various situations. These gases have to be monitored such that increase in the normal level of them could be known and proper precaution measures can be undertaken. So, an embedded system is designed using a microcontroller with internet of things, for the purpose of detecting and monitoring the hazardous gas leakage, which aids in the evasion of endangering of human lives. The hazardous gases can be sensed and displayed each and every second, in proximity to one more sensor for tracking heart beats which help to monitor the condition of the sewer labourers. If both the gases along with a pulse detector exceeds the normal level then an alarm is generated immediately and also an alert warning message can be sent to the authorised administrator and as well to the nearest health center to make the sewer labourers feel comfortable with necessary first aid and possibilities with the treatment in the case of emergency. Once the message is received by the health center, they enforce their team with necessary first aid to the current location to save the sewer labourer. Once this system is established for a particular user this will completely become fully automated and does not need any other additional people for monitoring and alerting purpose. It has an advantage over the manual method in offering quick response time and accurate detection of an emergency.

## 1 Introduction

The concept of gas detection is to detect the harmful gases that are present in the mixture of gases from the surroundings. The gas detectors are used to find the combustible, toxic and flammable gases. Earlier the gases were detected by using semiconductors, oxidation, catalytic, infrared etc. In common the gas leaks may occur in gas pipelines, domestic gas cylinders. In general, the various sensors used include infrared point sensors, ultrasonic sensors, semiconductor sensors and electrochemical sensors. The portable detectors are used in monitoring the air composition around the personnel and it can be worn on the personnel's belt, clothes etc. There are other types of sensors that are fixed sensors, they are basically mounted near the operation around the plant or control room. The gases are detected by the calibration of the sensors. All the gas sensors must be calibrated on a schedule. Pure natural gas is colourless and odourless and it is primarily composed of methane. Sewer gas is a complex mixture of toxic and non-toxic gases that are produced in sewage systems by the decomposition of organic matter. The gases that are present in the sewer gases may include gases like methane, carbon monoxide, hydrogen sulphide.

Since there is a need for a device that is with low power consumption along with more reliability and detection of the gases, the approaches like principal component analysis (PCA) and principal component regression (PCR) technique [1] are used to achieve low cost and low power consumption devices that are developed and heat loses are also reduced. Particular types of gases can be detected by using the metal–oxide–semiconductor technique, optic methods and acoustic methods [2] are used to identify four gases using a single sensor which can trace small amount of gases. The gas presence can also be detected by the chemically infused paper that changes its colour when exposed to gas by implementing ARM7 processor and by implementing Keilsoftware [3] to send alerts via Internet of things (IoT) with emergency turnoff power the system that is efficient as it has a low cost to detect LPG leakage in homes. While in industries the gas leakage is based on the physical phenomenon of absorption of infrared energy by combustible gases such as methane, propane or ethane which can be detected by a gas detector [4] which converts in the form of voltage, this voltage level is converted by an analogue-to-digital converter in digital form and this signal is the input for the microcontroller which displays the output. The importance of gas sensing is set to grow with increasing requirements for the need for safety and environmental protection across many industries.

The increasing number of deaths (fatalities) among miners caused by toxic gases in the mining industry requires an innovative approach to rescue the miner's health which is implemented by using an autonomous remote monitoring framework of wireless sensor networks [5],which integrates ohm's law and mobile computing with intelligence, hence governing decision making for miners. In few plants, though there is a monitoring system they not having the facility of continuous monitoring, which can be achieved by using LABVIEW [6] the message was sent by a global system for mobile communications (GSM) module which sends harmful gases emission level from the industries to pollution control board. There exists volatile gases which can evaporate easily soon known as volatile organic compounds which can be dangerous at various levels of concentration levels in the air, which can be detected by using an electronic nose [7] that consists of three different sensors that can detect gases on various heater voltages which gives an accuracy rate of 100%. The system lacks the usage of more no. of sensors.

There exists a method to determine the amount of toxic gases by using the current generated by electrochemical cells or catalytic sensors, the new method will detect the gas leakage using the acoustic wave [2, 8] which deploys highly sensitive miniature micro-electro-mechanical system microphones and it uses a suite of energy-decay and time delay of arrival algorithms which are used for localising a source of gas leak with low power consumption and scalability. There is a huge demand for intelligent portable distributed environmental monitoring and control system for detecting the greenhouse gases, which can be detected by using three different detachable sensors [9] that have been developed along with smart transducer interface module. As the proposed model shown in Table 1 has more advantages over existing models in terms of gas sensors used, technology implemented, air contamination identified, calibration stages and time taken. This proposed methodology has optimised the time on the basis of the microcontroller algorithm. Various gases are released from the oil wells which may be harmful to workers through inhalation or absorption through the skin, hence solid state sensors [10] and wireless sensor node communication [11]. A classification was introduced for different types of gases and their odours known as slope multiplication with dynamic features of thick film gas sensor array [12]. An exploration of pattern examination of machine smell capability was implemented by a member of IEEE [13]. A contemporary feature called mean slope multiplication to measure each gas and its odours using powerful responses of the sensor array [14]. A soft computational approach using multi-scale principle component analysis for bigotry of gases was presented, which was found to identify gases with high probability. Hence system will be compact in size with low power consumption, the system not only detects the gas leaks but also detects the concentration of the gases and it rings an alarm when the level goes beyond the limit. The main origin of this idea was due to the events that have occurred lately on the people who had entered the sewers [15, 16].

**Table 1:** Comparing the existing model with the proposed system

Authors	Gas sensors applied	Technology implemented	Air contamination identified	Calibration stages	Time taken
S. Capone and P. Siciliano	CH_4_ and CO	Integration and fabrication	CO, NO_2_, CH_4_, and SO_2_	PCR	54 ms
S. Sudharshanan and C. Balasundar	MQ-7	GSM, TX-RX mode	NO*_x_* and CO	Binary logic	65 ms
Sunny Sharma and V.N. Mishra	E-nose	Neural network architectures (NNAs): NNA1 and NNA2	CO, NO_2_, and CH_4_	activated sludge mode (ASM) method	43 ms
Isaac O. Osunmakinde	CH_4_ and CO	Long-distance wireless sensor network	NO_2_, H_2_S, and CH_4_	Ambient intelligence	44.3 ms
|D. Bhattacharjee and R. Bera	Barometric and alcohol	GSM, TX-RX mode	N_2_O, CH_4_ and CO_2_	detachable smart transducer interface module (DSTIM)	60.23 ms
K. PadmaPriya and M. Surekha	MQ 5	Firmware, GSM, TX-RX mode	CH_4_, CO, and NO_2_	Looping control	37.3 ms
Ricardo Gutierrez-Osuna	metal oxide semiconductor (MOS), conducting polymer (CP)	Pattern analysis, feedback analysis	N_2_O, CH_4_, SO_2_	PCA, laser Doppler anemometry (LDA), frequency selective surface (FSS)	29.43 ms
Proposed method	MQ 2, MQ 4, MQ 6, MQ 7B	IOT,GSM,TX-RX mode, artificial neural network (ANN), automation etc.	Methane, propane, carbon monoxide, NH_3_, benzene, some general gases etc.	Binary logic, Marley model calibration	15.50 ms

## 2 Describe design interventions in the proposed system

**Fig. 1:**
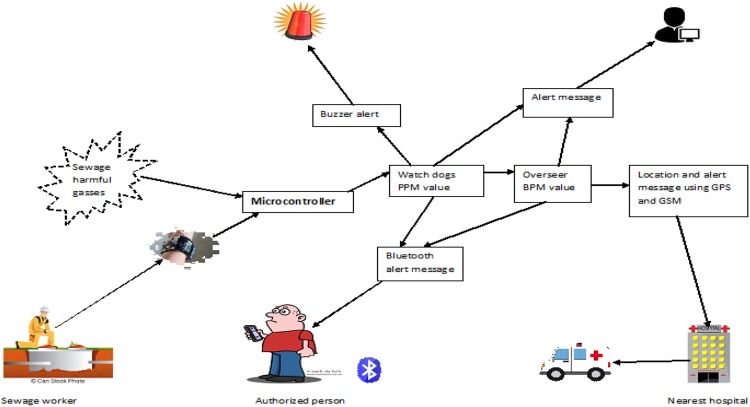
System structure

In developing countries like India, sewages are still cleaned by people. Hence this leads to exposure of those humans to hazardous gases such as methane, carbon monoxide etc. This system connected through IoT is developed to monitor the levels of these gases continually when the people enter the sewer. We take two values into consideration – parts per million (ppm) and beats per minute (bpm). Each value is uploaded to the database which is compared with reference data. The reference data contains the normal data of the hazardous gas and the normal rate of the bpm when the user is in action. The sample data are given below, which are dependent on the age of each person. In the database, the reference data are segregated based on the different age of the person. The basic idea is to determine a safe limit point ‘*x*’, when the system detects a value ‘*y*’ (near to or less than *x*) which then leads to alerting the people. If the value exceeds ‘*x*’ then a quick high alert is given for all to evacuate the premises. For calculation of bpm, a heartbeat sensor is worn on the wrist by each individual which continuously monitors the pulse rate with each and every value of the reference data. There are various levels of bpm that can be classified as dangerous or normal. These two main calculated values integrate with each other and if the monitoring data show major deviation, then the system finally comes to a conclusion on the level of danger in the environment. The overall system can be split into five phases: (i) evaluation of ppm value; (2) evaluation of bpm value; (3) uploads the data to monitor; (4) compares with the reference data; (5) alert sending system (Fig. 1).

### 2.1 Evaluation of PPM value

Arduino helps to detect the level of gas which is displayed on the LCD display. The whole system is connected through IoT, sends the data to the database and compares with the reference values. Once the level goes beyond a point, the system sends the alert message to a nearby health center [17].

### 2.2 Evaluation of bpm value

It is a wearable device which can be worn on the wrist. This helps to monitor the bpm level of the person. It works on the principle of the photoplethysmography, in which the change in the blood flow will also change the light intensity of the vascular region. This device sends the light rays to the blood cells, as the blood volume changes it also change the reflected light intensity. On processing the light intensity we can derive the bpm of the person. This device can be worn on the wrist or the pinnacle of the ear.

This sensor consists of a transmitter (Tx) and a receiver (Rx). The transmitting section consists of a light emitting device, and the Rx section consists of a photo-diode for detecting the light rays.

The heart beat pulses will cause the variation in the flow of blood in various regions of the body. When the tissue is illuminated with the light source, light emitted by the led, it may either reflect or can transmit the light, some of the light is absorbed by the blood and transmitted or can be reflected light received by the light detector. The amount of the light absorbed depends on the blood volume in that tissue. The detector output is in the form of electrical signal and is proportional to the heart beat rate. This signal is actually a direct current (DC) signal relating to the tissues and the blood volume, and the alternating current component synchronous with the heart beat which is caused by pulse changes in the arterial blood volume is been superimposed on the DC signals.

When it comes to implementation, the microcontroller data pins can be connected based on what type of input we depend on such as the analogue signal as well as the digital signal. In this proposed method, we connect the sensor to analogue pins of the Arduino. This microcontroller takes the analogue value of the sensor which sent the value through IoT to the database; these values are plotted and compared with the reference data periodically without any time delay variations. During the deviation, the MySql checks for the variations in the heart rate in parallel. In both the cases if any one of the cases shows major deviations, the alert message will be sent to the user and the authorised person (Tables 2 and 3).

**Table 2:** Reference data in the database which is segregated based on the age with critical heart beat rate of a sewage worker during the cleaning process

Age	Critical heart beat range		Target heart beat zone
	Tachycardia	Bradycardia	
below 20 years	>120 bpm	< 90 bpm	100–170 bpm
20–30 years	>100 bpm	< 85 bpm	95–162 bpm
30–40 years	>150 bpm	< 70 bpm	93–157 bpm
40–50 years	> 130 bpm	< 80 bpm	88–149 bpm
50–60 years	> 145 bpm	< 75 bpm	85–145 bpm
60–70 years	> 90 bpm	< 60 bpm	80–136 bpm
70–80 years	>110 bpm	< 80 bpm	75–128 bpm

**Table 3:** Symptoms related to the respective beat zone (ANN-calibration)

Bpm zone, bpm	Symptoms
>159	stomach pain, lightheaded, snoring
>166	throat or jaw pain, irregular heart beat
>182	chest discomfort, indigestion, heartburn
>179	stomach pain, throat or jaw pain
>186	chest discomfort, heartburn, lightheaded
>169	nausea, lightheaded, irregular heart beat
>151	chest discomfort, indigestion, heartburn
>137	nausea, heartburn
>133	stomach pain, irregular heart beat
>130	chest discomfort, indigestion, heartburn
>119	throat or jaw pain, snoring irregular heart beat

T1= time difference between the first two heart beat (in ms)

T2 = time difference between the next to the two heart beats (in ms)

*k***=** T1**–**T2,

*m* = (*k*)/5,

*n* = 60,000/m,

Bpm rate, where *k* is the time taken for the five pulse |; *m* is the time for the single pulse time; *n* is the total bpm rate.

**Fig. 2:**
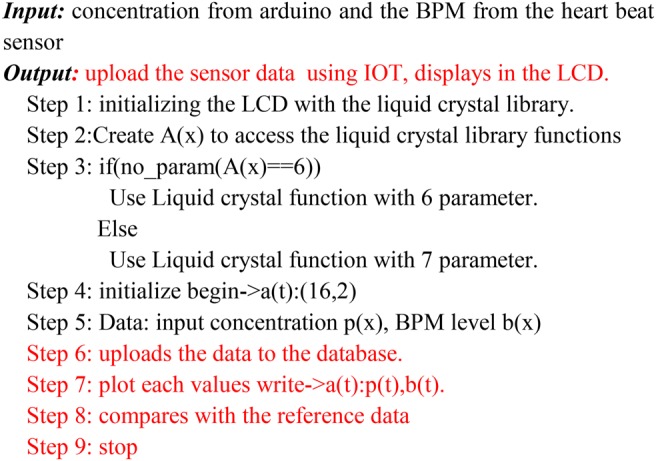
Algorithm 1: Display the concentration of gases and the bpm level of the person

The pulse of the person is unique. Which can vary from person to person as the age of the person is depended on it. To find the exact processed data we have to use the below procedure:

The rate of the bpm = 211−(0.64 × age) (see Fig. 2)

### 2.3 Alert sending system

**Fig. 3:**
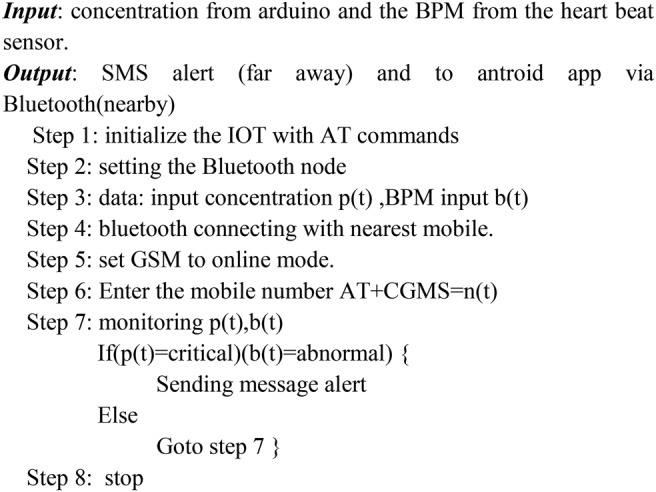
Algorithm 2: Provide alert to the user along with his/her bpm and effect

The microcontroller processes all the input data from the sensor. When the gas level becomes critical, a buzzer is available to alert the user about the danger of the environment. When the bpm level of the worker reaches a perilous level, the GSM module will put the overall system to online and sends the emergency alert message to the worker, the administrator and the nearby emergency health center for taking immediate action on the situation (see Fig. 3).

**Fig. 4:**
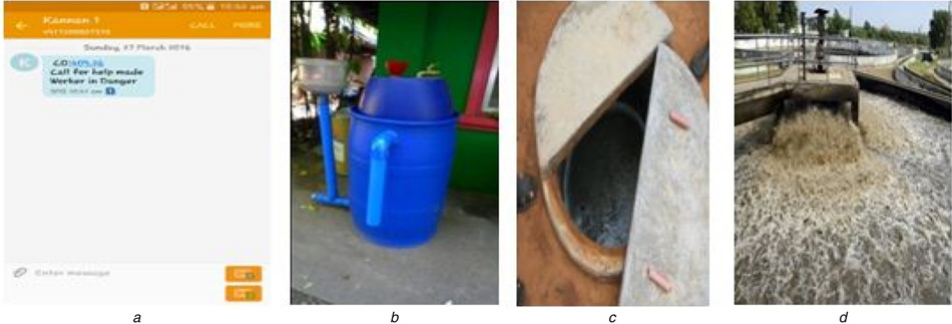
Various sources of hazardous gases and alert system *a* Alert message from the proposed system *b* Biodigester *c* Septic tank of an organisation *d* Waste water treatment plant

Fig. 4*a* shows the message obtained from the proposed system. It expresses the ppm level of the specific gas that has been recognised by the system in the current scenario.

## 3 Experimental results

The developed system was able to produce a response within 10–20 s. Most of the traditional gas systems require 25 V power supply. However, this system reduces power consumption, requiring only 5 V of power supply that can be provided by a battery. The lifetime of the sensors is nearly 4–5 years up to which it remains stable. Due to low power consumption, minimum heat is generated. The system is designed to work with high optimisation under conditions like high temperature and humidity as in that of south Indian region. The concentration of the gases calculated using this system is more accurate when compared with other systems. The deviation in concentration was 4% less in carbon monoxide and lesser than 10% in methane for a lower concentration of gases.

### 3.1 Test for methane and carbon monoxide using bio-digester

Methane is a highly flammable gas with a low energy density that is easy to produce. In fact, methane is produced by man on a daily basis. The key to produce methane is bacteria.

The production of methane consists of a large tank or digester shown in Fig. 4*b* (where methane is produced), a temperature control, an agitator or stirrer, and a gas storage tank. Methane can be made from anything, and it is because of this that it is a common product of many farms around the world. Waste from other bio-fuel processes can be used, as well as excreta from humans and animals, food wastes, agriculture residues, and just about anything that was living at one time or another.

Mixing the feedstock with some water and keeping the oxygen away, is likely to produce biogas without much effort. The amount of biogas produced depends on the feedstock and its composition. If you are looking for a carbon to nitrogen ratio of about 30:1, then that is 30 browns to each green (by weight), which coincidentally is the perfect ratio for an aerobic compost heap. The continuous feed system is sized such that the daily input is digested over 30–60 days. Water will have to be input daily, as well so, if you could reasonably reuse some of the water that is discarded daily, this would increase the efficiency of the system. Temperature plays a big role in the formation of methane. Bacteria thrive in the bodies of animals, so they evolve to survive in warm, moist, zero oxygen environments. The most efficient temperature range is 90–110 °F (32–43°C). So, to most efficiently use dung, a heating system is required, or at the very least there must be a way to keep the temperature constant. This is the best done with a good tank insulation and a solar water heater. As a backup, some of the methane could be burnt to keep the tank warm. Once the gas is produced, it needs to be collected and stored somewhere. It is fairly simple to make a water float gas tank. Methane can be stored in large, low pressure containers which are safe and also easy to construct. However, in the interest of space and energy density, it can also be compressed. However, care must be taken to avoid mixing of oxygen with the compressed methane because that can lead to explosion. Compressed methane can be used in vehicles (compressed natural gas), or can be used like natural gas, or propane in stoves or other gas appliances. By using a simple, PVC pipeline the produced gas can be efficiently connected to appliances. Since it is highly inflammable, some safety precautions should be made like pressure gauges, gate-valves to close the pipeline etc.

**Fig. 5:**
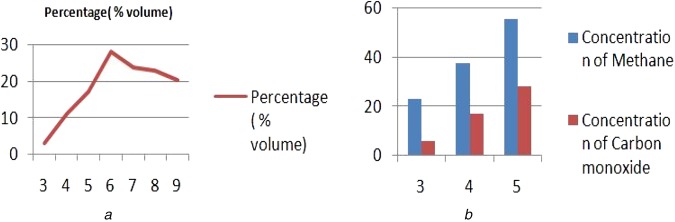
Variation of the gases over time *a* Variation in methane concentration with time *b* Variation in concentration of two gases over time

The concentration of methane does not remain constant. It increases initially until it reaches peaks, and then slowly starts reducing in volume. The peak percentage occurs approximately at around day 6. The percentage of rise and fall is illustrated graphically in Fig. 5*a*.

### 3.2 Test conducted in septic tank of an organisation

In an educational institution, human urinal wastes were collected and then the purification process was carried out. Testing was done at the septic tank opening and at the places of various stages of the purification process. We analysed that the concentration of the methane and carbon monoxide differ from one place to another. For example, the concentration of both the gases is higher at the brink of a tank. The concentration of the gases starts decreasing when the urinal wastes have undergone various purification stages. The concentrations at various stages are shown below in Tables 4 and 5.The concentrations of methane and carbon monoxide were compared over the duration of three days. Fig. 5*b* depicts how they consistently rose in their respective volumes, with the progression of days. The concentration of methane was greater than the concentration of carbon monoxide in all instances.

**Table 4:** Concentration of methane at various times

S. No.	No. of days	Percentage, % volume
1	3	3.0
2	4	11.1
3	5	17.1
4	6	28.1
5	7	23.8
6	8	22.8
7	9	20.2

**Table 5:** Concentration of methane and carbon monoxide at various days

S. No.	Days	Concentration of methane	Concentration of carbon monoxide
1	3	23.2	6.0
2	4	37.5	17.2
3	5	55.6	28.1

### 3.3 Testing conducted in a waste water treatment plant

All municipality wastes from houses, offices and various places were dumped in a place. Then the water was treated in various stages using different purifying methods. Later, the treated water was used to water plants in public parks. The concentration of the methane and carbon monoxide was measured during various filtration processes. It was verified that the emission of methane and carbon monoxide from the waste water was not as much high as emitted from the septic tank. The concentration of the gases seemed like it did not cause any major effects to the human. However, it led to various human health hazards when exposed to, for a long time (Table 6).

**Table 6:** Comparisons of the overall experimental results

Experiments	Temperature, f	Concentration	Experimental durations
a test for methane and carbon monoxide using a bio-digester	90–110	23.43	7 days
testing conducted in a waste water treatment plant	60	45.34	4 days
test conducted in septic tank of an organisation	86	56.98	6 days

## 4 Conclusion

We have proposed a system for identifying gases that cause death using a wireless sensor system. The system has high accuracy because of the calibration procedure that is followed. The system is able to detect multiple gases at the same time. The sensor data are uploaded to the database continuously, each value is compared with the reference data. If there is any major deviation, then the system alerts the user depending on the levels of the hazardous gases. The system can be modified according to the environmental conditions. The system gives the user warnings, along with the health hazards that may be caused due to continuous exposure to the gas at a particular concentration. There is also a monitoring system which is used to track the heartbeat of a labourer. By using IoT, an alert message will be sent to the proximal health center for rescuing the labourer in the case of emergency. A minor limitation of this research is network reliability. The writing of the letter using latest papers is relevant and the ideas of implementation are exemplary. The conclusion and further progress in this research are obvious and more helpful for the society in handling hazardous gases.

## 5 Funding and declaration of interests

None declared.
